# 3D-printed hydrogels dressings with bioactive borate glass for continuous hydration and treatment of second-degree burns

**DOI:** 10.36922/ijb.0118

**Published:** 2023-07-14

**Authors:** Fateme Fayyazbakhsh, Michael J. Khayat, Candy Sadler, Delbert Day, Yue-Wern Huang, Ming C. Leu

**Affiliations:** 1 Department of Mechanical and Aerospace Engineering, Missouri University of Science and Technology, Rolla, Missouri, USA; 2 Intelligent System Center, Missouri University of Science and Technology, Rolla, Missouri, USA; 3 Center for Biomedical Research, Missouri University of Science and Technology, Rolla, Missouri, USA; 4 Department of Materials Engineering, McGill University, Montréal, Quebec, Canada; 5 Wound Clinic, Phelps Health Hospital, Rolla, Missouri, USA; 6 Department of Material Science and Engineering, Missouri University of Science and Technology, Rolla, Missouri, USA; 7 Department of Biological Sciences, Missouri University of Science and Technology, Rolla, Missouri, USA

**Keywords:** Hydrogel wound dressing, Burn wound healing, 3D printing, Bioactive borate glass, Continuous water release

## Abstract

Recent advances in additive manufacturing have led to the development of innovative solutions for tissue regeneration. Hydrogel materials have gained significant attention for burn wound treatment in clinical practice among various advanced dressings due to their soothing and moisturizing activity. However, prolonged healing, pain, and traumatic removal due to the lack of long-term wound hydration are some of the challenges in the treatment of second-degree burn wounds. In this study, 3D-printed dressings were fabricated using gelatin, alginate, and bioactive borate glass (BBG) using an extrusion-based bioprinter. After ionic crosslinking, the 3D-printed dressings were characterized for mechanical properties, degradation rate, hydration activity, and *in vitro* cell viability using human fibroblasts. The results demonstrated that in 3D-printed dressings with 20 wt% BBG, Young’s modulus increased by 105%, and 10-day degradation rate decreased by 62%. Addition of BBG prevented the burst release of water from hydrogel dressings and enabled the continuous water release for up to 10 days, which is crucial in treating second-degree burn wounds. 3D-printed hydrogel dressings with BBG showed long-term cell viability that can be a result of the accumulative release of therapeutic ions from BBG particulate. The *in vivo* wound healing functionality of the dressings was investigated using a rat model with a second-degree burn wound. Our animal study showed that the 3D-printed dressings with BBG exhibited faster wound closure, non-adhesive contact, non-invasive debridement, and non-traumatic dressing removal. Histological analysis suggested that 3D-printed dressings contributed to more uniform re-epithelialization and tissue remodeling compared to the non-printed hydrogels of the same compositions. Critically, 3D-printed dressings with BBG led to significant regeneration of hair follicles compared to the 3D-printed hydrogel, non-printed hydrogel, and the control groups. The superior outcome of the 3D-printed hydrogel–BBG20 dressings can be attributed to the bioactive formulation, which promotes moist wound healing for longer time periods, and the non-adhesive porous texture of the 3D-printed dressings with increased wound-dressing interactions. Our findings provided proof of concept for the synergistic effect of bioactive formulation and the porous texture of the 3D-printed hydrogel dressings incorporated with BBG on continuous water release and, consequently, on second-degree burn wound healing.

## 1. Introduction

Burn wound healing is a complex and delicate molecularcellular process to restore skin functions and repair tissue damage^[1]^. Burn injuries represent a major public health issue and are among the most severe injuries^[2]^, with approximately 10,000 annual deaths in the United States^[3]^. Each year approximately 1.1 million patients suffering from burn wounds are admitted to hospitals in the United States, and approximately 660,000 among them are diagnosed with second-degree burn wounds^[4–6]^.

Second-degree burn is characterized by damage to the integrity of the entire epidermis and varied depths of the dermis that typically require immediate medical care^[7]^. The current standard of care in clinical practice for second-degree burn wound treatment includes topical antimicrobial agents and advanced dressings such as contact dressing and hydrogel dressings. The primary goal of the advanced dressings is to promote optimal wound healing while providing pain relief and protection from infection^[8,9]^. Although the existing advanced wound dressings cover a broad range of moderate to high exuding wounds, a small number of wound care products are specifically designed for low exuding wounds, *e.g*., second-degree burns. Despite good clinical outcomes for wet wounds, the majority of these products end up with inadequate healing and poor hydration in second-degree burn wounds^[10,11]^. Topical agents, such as silver sulfadiazine, are typically associated with pain and prolonged healing due to wound dehydration, poor re-epithelialization, traumatic removal, and non-transparent appearance^[10–22]^. Hydrogel dressings provide a moist environment for the second-degree wound and help with pain relief. However, frequent changes of hydrogel dressings in second-degree burn wounds can be time-consuming, potentially traumatic to the healing tissue, and may lead to complications such as dehydration or maceration if poorly managed. Hence, there is an immense need to develop advanced wound dressings for second-degree burn wound treatment with desired features, such as bioactive formulation, soothing effect, tunable water absorption/release, non-adhesive contact, and skin-like mechanical properties^[6,16,23,24]^.

Bioactive glass materials are a class of bioceramics that have the ability to bond with living tissues and promote specific biological responses when exposed to body fluids. These materials have been studied extensively for their potential use in hard tissue engineering and implant coating^[25,26]^. Bioactive glass materials comprise a mixture of oxides from biologically active elements, such as Si, B, Ca, Mg, Ag, Ce, Cu, and Zn, among others. The multiple therapeutic ions released from bioactive glass can react with proteins and enzymes to stimulate the proliferation and differentiation of cells involved in wound healing, such as mesenchymal stem cells, fibroblasts, and endothelial cells^[26,27]^. There are three major types of bioactive glass which are classified based on the dominant network forming oxide in the glass formulation: silicate glass, phosphate glass, and borate glass, among which the latter has the fastest degradation rate^[28]^. Recently, the US Food and Drug Administration (FDA) has cleared a bioactive borate glass (BBG) product (Mirragen, ETS Wound Care, Missouri, USA) for treatment of chronic wound healing^[29]^. In addition to its ability to promote cellular activity, BBG can enhance wound healing by (i) regulating the secretion of essential growth factors and matrix metalloproteinase (MMPs) to promote angiogenesis and extracellular matrix (ECM) remodeling^[30–37]^, (ii) preventing bacterial growth^[32,38,39]^, and (iii) alleviating the adverse inflammatory response by scavenging the reactive oxygen species^[40,41]^. Several studies showed that boron in BBG positively affects different stages of wound healing. It has been shown that boron can promote angiogenesis by stimulating tissues to secrete specific growth factors and modulate keratinocytes and endothelial cellular responses^[26,31,36,42,43]^. One of the key advantages of using BBG over other additives is its potential for incorporating a wide variety of dopants, including copper and zinc to promote the release kinetics of bioactive ions, regulate degradation rate, and potentially impart additional therapeutic properties to the glass^[44]^. Copper has been shown to stimulate the proliferation of endothelial cells by inducing local hypoxia to stimulate the expression of vascular endothelial growth factor (VEGF)^[33,45]^. Several studies have reported the positive effect of zinc dopant on the antimicrobial activity and inflammatory response^[46–48]^.

Despite the good clinical outcome for wet wounds, however, BBG alone is ineffective in treating dry wounds, *e.g*., burn wounds, due to the wound dehydration and lack of reacting medium. Besides, the fast degradation rate and ion release from BBG make it highly reactive, which can damage the fragile wound bed in the case of burn wounds^[49]^. On the other hand, the burst release of therapeutic ions from BBG can increase the local pH to alkaline range, which can cause local toxicity and tissue damage. An effective approach to address this challenge is to incorporate BBG particulates in natural hydrogel matrices. The BBG–hydrogel complex can take advantage of the synergistic activity of therapeutic ions from BBG and the soothing effect of the hydrogels, making it effective for burn wounds. While the hydrogels provide water for wound hydration, BBG particulates prevent the burst release of water molecules from the hydrogel network, making it stable on the wound surface for longer time periods.

Hydrogels are large 3D molecules composed of hydrophilic polymer chains that absorb and retain large amounts of water^[50]^. Hydrogels are an essential class of advanced wound dressings that can donate or absorb water in accordance with the wound condition^[7,51,52]^. Since 1977 when hydrogels were introduced as wound dressings for the first time^[53]^, their biological performance has been enhanced by adding glass materials, peptides, and growth factors^[54–56]^. In 2019, Zhu *et al*. incorporated ZnO/silicate glass and epidermal growth factor (EGF) into alginate/chitosan hydrogel compound. Their results showed that the ZnO/silicate glass promoted the formation of granulation tissue, deposition of collagen and myofibril, release of anti-inflammatory factors, angiogenesis, and wound closure^[57]^. In 2020, Zhu *et al*. showed that silicate glass/alginate compound can modulate the inflammatory response and angiogenesis^[52]^. Chen *et al*. incorporated GelMa with cerium-doped silicate glass to develop an injectable compound for treatment of diabetic ulcers. Their results showed that cerium-doped glass improved the angiogenesis and antibacterial activity of the hydrogel by regulating the anti-inflammatory response^[28]^. In another research published in 2022, Mehrabi *et al*. developed *in situ* forming hydrogel dressing by incorporation of borate glass into chitosan/carboxymethyl cellulose hydrogel compound. Their results showed consistent angiogenesis, remodeling, and accelerated wound healing in diabetic rats^[58]^. Although the use of novel hydrogels incorporated with bioactive glass has paved the road for effective wound treatment, the majority of these hydrogel dressings are amorphous gels with poor mechanical stability on the wound and short-term wound coverage. These shortcomings may result in frequent change of wound dressing that cause pain and infection.

3D printing, as a rapidly developing manufacturing technology, enables the programmable and customizable high-throughput fabrication of wound dressings with multiple materials and geometries^[59]^. Our previous research showed that 3D-printed dressings with an adequate gelatin/alginate ratio promote burn wound healing^[51]^. We highlighted that the 3D-printed porous surface supports non-adhesive contact and increases the available surface for wound-dressing interactions compared to non-printed dressings of the same composition. However, wound dehydration remains a major clinical challenge in burn wound treatment. In the research, we incorporated 3D-printed hydrogel dressings with BBG, which allows for controlling the release of water and therapeutic ions as well as enhancing the dressing fixity on the wound for longer time. To the best of our knowledge, the current research investigates the use of 3D-printed glass-hydrogel wound dressings for the first time.

In this study, 3D-printed dressings with gelatin, alginate, and various amounts of BBG were fabricated and characterized to enhance the functionality of burn wound care products. After measuring the mechanical properties, degradation rate, hydration activity, water release rate, and *in vitro* biocompatibility, an *in vivo* wound healing study was conducted to investigate the effect of BBG and 3D-printed porous contact on treatment of second-degree burn wounds using a rat model. BBG powder, non-printed hydrogels of the same formulation, and a commercial product were included in the experiment.

## 2. Materials and methods

### 2.1. Materials

Gelatin type B (from bovine skin, gel strength: 225 g Bloom), sodium alginate (alginic acid sodium salt from brown algae, medium viscosity), calcium chloride anhydrous, Dulbecco’s Modified Eagles Medium (DMEM), phosphate-buffered saline (PBS), fetal bovine serum (FBS), 1% penicillin/streptomycin (pen/strep), MTT, and trypsin/EDTA were purchased from Sigma-Aldrich (St. Louis, Missouri, USA). BBG (particle size <20 μm) was provided by ETS Wound Care (Missouri, USA). All materials were used as received without further modification.

### 2.2. Bioink preparation

**Figure 1.**
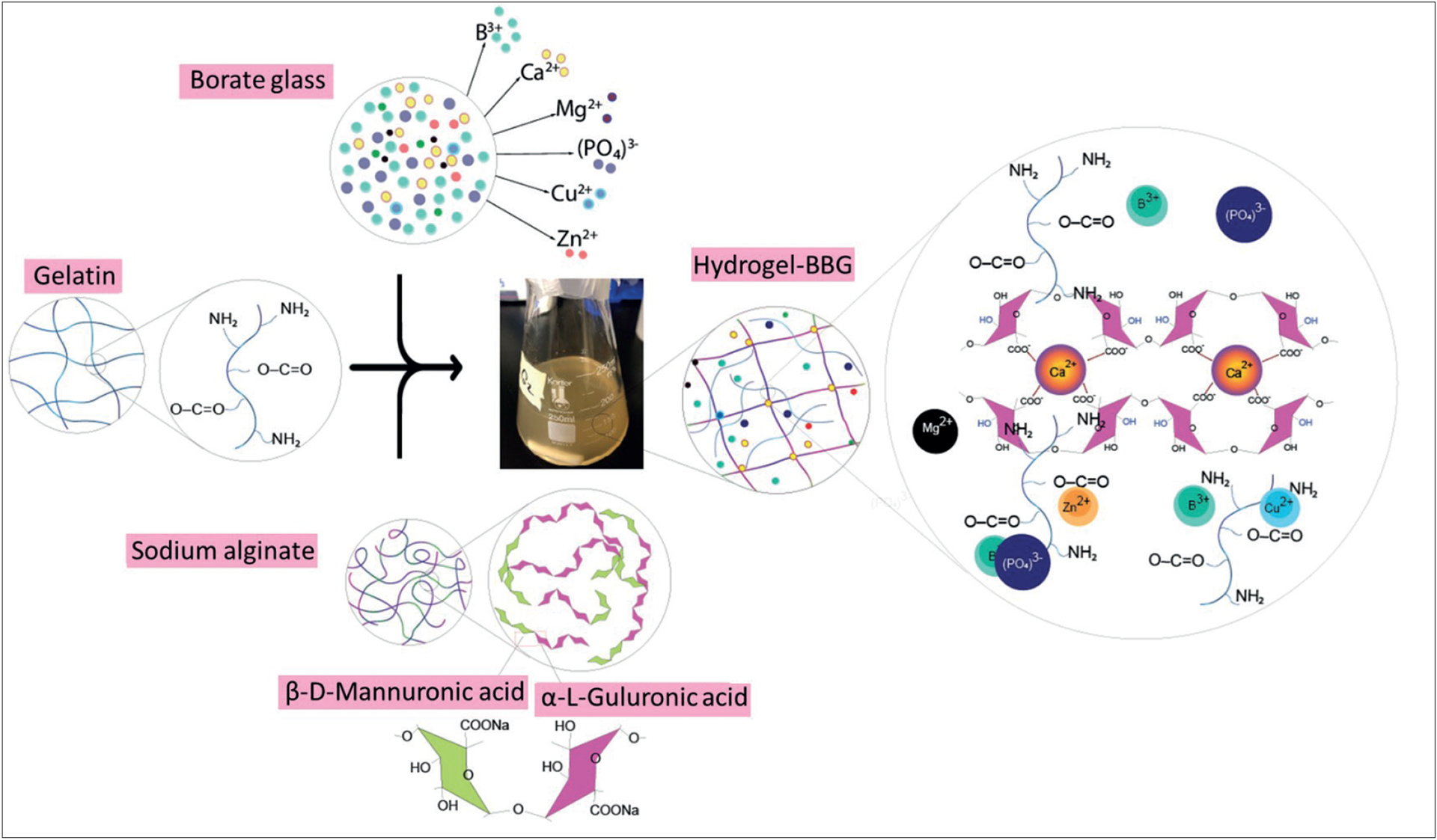
Schematic of the structure of gelatin, BBG, sodium alginate, and hydrogel–BBG blend. Gelatin and alginate are semi-interpenetrating networks (semi-IPN), whereby the free volume decreases due to (i) the electrostatic interactions between BBG particulates and gelatin–alginate chains and (ii) the formation of crosslinks between alginate chains.

In this study, the gelatin:alginate ratio of 5:3 was selected as the primary hydrogel matrix based on our previous findings, which demonstrated the optimal balance in terms of mechanical properties, printability, and biocompatibility in this hydrogel compound^[16]^. To prepare the hydrogel–BBG bioinks, different concentrations of BBG (0, 10, and 20 wt% of dry material) were mixed with 7 mL of deionized (DI) water and vigorously stirred for 24 h at room temperature. After centrifuging and filtering, the resulting supernatants were mixed with 500 mg of gelatin powder and stirred for 10 min at 40°C to obtain 5% (w/v) gelatin, gelatin-BBG10, and gelatin-BBG20 solutions. A 10 w/v% stock solution of sodium alginate was prepared by dissolving 1000 mg of sodium alginate powder in 10 mL of DI water. To achieve a gelatin:alginate ratio of 5:3, 3 mL of the sodium alginate stock solution was added dropwise to the gelatin–BBG solutions and stirred at 800 rpm for 40 min at 40°C to obtain clear homogeneous compounds of hydrogel, hydrogel–BBG10, and hydrogel–BBG20 bioinks. The schematic microstructure of gelatin, alginate, BBG, and their mixture is shown in Figure 1.

### 2.3. 3D printing

In this research, extrusion-based 3D printing technology was utilized using the Inkredible^®^ bioprinter (CELLINK Corporation, Sweden). The dressings were printed directly onto sterile Petri dishes with the print head temperature adjusted at 25°C and 35°C for hydrogel and hydrogel–BBG bioinks, respectively. The dressings were printed at 2.5 mm/s speed and 100 kPa pressure with a geometry of square (30 × 30 × 3 mm^3^) and dog bone (30 × 10 × 5 mm^3^) for different tests. The 3D-printed dressings were immersed in 0.2 M calcium nitride (CaNO_3_) solution for 10 min to form crosslinks between alginate chains. After crosslinking, 3D-printed dressings were rinsed with DI water three times and stored at 4°C.

### 2.4. Mechanical testing

The dog-bone-shaped dressings were tested for mechanical properties using the Universal Instron 5969 Dual Column Testing System (Instron, Massachusetts, USA) and the BlueHill Universal Software (*n* = 5). The scaffolds were assessed using a uniaxial tensile load frame at 5 mm/min, typical for polymer specimens to measure the modulus of elasticity, yield strength, and yield strain of the scaffolds in accordance with the ASTM F2150–8 standard.

### 2.5. Evaluation of chemical structure

The chemical structures of the bioinks were identified using a Nicolet iS50 Fourier-transform infrared spectroscopy (FTIR) spectrophotometer (Thermo Scientific, Massachusetts, USA) equipped with a diamond crystal cell of attenuated total reflection (ATR) accessory. All the spectra were recorded at a resolution of 4 cm^−1^ with 32 scans with a data spacing of 0.482 cm^−1^ in the mid-infrared region (4000–400 cm^−1^). The obtained spectra were analyzed with OMNIC 9.2.41 software (Thermo Scientific, Massachusetts, USA). The infrared (IR) spectrum data from Sigma Aldrich were used to identify characteristic chemical bonds in gelatin, alginate, and water.

### 2.6. Swelling capacity and biodegradation rate measurement

The swelling capacity and degradation rate of the 3D-printed dressings were measured by measuring the gravimetric changes of the samples after immersing in PBS. The samples were weighed, immersed in PBS, and kept at 32°C to reach equilibrium swelling and subsequent degradation. The weight changes in determined time intervals were recorded for up to 7 days as the dressings are intended to stay on burn wounds for up to 7 days (*n* = 5). The dressing’s swelling capacity and degradation rate were calculated using the following equations:

(I) $$\text{Swelling}\;\text{capacity}(\%)=\frac{{\text{W}}_{\text{max}}-{\text{W}}_{\text{Dry}}}{{\text{W}}_{\text{Dry}}}\times 100$$

(II) $$\text{Degradation}\;\text{rate}(\text{mg}/\text{min})=\frac{{\text{W}}_{0}-{\text{W}}_{\text{day}10}}{10\times 24\times 60}$$

where *W*_Dry_ is the initial dry weight, *W*_max_ is the maximum weight of the scaffolds after immersion, and *W*_day10_ is the weight after 10 days of immersion in PBS.

### 2.7. Hydration activity and water release kinetics measurement

To measure the effect of BBG on the water release rate, the hydration activity was measured, as described in our previous work^[16]^. Briefly, the total amount of water in each sample was measured using thermogravimetric analysis (TGA) on 250°C for 10 min (SDT Q600 V20.9 Build 20, Universal V4.5A TA Instruments, Minnesota, USA). The weight change was considered the total water content (*n* = 5)^[60]^.

To predict the hydration activity of the samples on burn wounds, the water release rate from each sample was measured using an ethylcellulose super absorbent foam (Shield Line LLC, New Jersey, USA), as a model of the dehydrated burn wound. After placing the 3D-printed dressings on the foam surface, the gravimetric changes were measured after 24 h at 32°C, as the temperature of the burned area is often lower than the normal body temperature^[61]^. The 24-h hydration is a key factor in burn wound treatment outcome, as the systemic capillary leak, intravascular fluid loss, and significant fluid shifts mostly occur within the first 24 h, peaking at around 6–8 h after injury^[62,63]^. The total water content and water release rate were calculated using the following equations:

(III) $$\text{Total}\;\text{water}\;\text{content}(\%)=\frac{{\text{W}}_{0}-{\text{W}}_{\text{H}}}{{\text{W}}_{0}}\times 100$$

(IV) $$\text{Water}\;\text{release}\;\text{rate}(\%)=\frac{{\text{W}}_{0}-{\text{W}}_{24}}{{\text{W}}_{0}-{\text{W}}_{\text{H}}}\times 100$$

where *W*_0_ is the initial weight, *W*_*H*_ is the weight after heating at 250°C, and *W*_24_ is the weight after 24 h placing on dry surfaces.

The water release profile was calculated and plotted against time (up to 10 days). To determine the water release kinetics, the release profile was analyzed using different kinetic models, including zero-order, first-order, Higuchi, Korsmeyer–Peppas, and Hixon–Crowell for each dressing formulation^[64]^.

### 2.8. MTT assay

The MTT assay was used to study the effect of BBG on the viability and proliferation of human dermal primary fibroblast cells (ATCC, Virginia, USA) at passages 3 and 4. 3D-printed dressings were weighed and exposed to ultraviolet light (345 nm) for 15 min per side, then immersed in DMEM with no further treatment. The sample extracts were collected and filtered after 1, 3, and 7 days of immersion (five replications). The sample extracts were used to indirectly evaluate the cell viability in accordance with the ISO-10993 standard. The DMEM culture media with no further treatment was considered the control sample. Cells were cultured in DMEM containing 10% FBS and 1% pen/strep until they reached 80%–90% confluence. Cells were seeded in 96-well plates at a density of 10^4^ cells/well with 100 μL of DMEM containing 10% FBS and 1% pen/strep. The plates were incubated at 37°C with 5% CO_2_. After 24 h, the initial culture media were discarded and replaced by 90 μL sample extracts with 10% FBS and 1% pen/strep. Following 24 h of treatment, 100 μL of 0.5 M MTT solution was added to each well and incubated for 4 h. Formazan crystals were solubilized using isopropanol, and after 30 min, the absorbance was measured at 545 nm using an ELISA reader (Stat Fax 2100, Awareness Technology Inc., Florida, USA).

### 2.9. Animal test

#### 2.9.1. In vivo burn wound model

All *in vivo* experiments were approved by the Missouri S&T Institutional Animal Care and Use Committee (IACUC) (Reference No. 177–20). The ability of the 3D-printed wound dressings for the treatment of second-degree burn wound was evaluated by creating a circular burn wound in the dorsal area of Sprague Dawley rats using a hot aluminum bar. Thirty-six Sprague Dawley rats (Charles River, Missouri, USA) were divided into six groups with six animals per group:

- Control: Wounds covered with petrolatum gauze as the current standard of care
- BBG powder: Wounds covered with BBG powder
- Non-printed hydrogel: Wounds covered with non-printed hydrogel
- 3D-printed hydrogel: Wounds covered with 3D-printed hydrogel dressings
- Non-printed hydrogel–BBG: Wounds covered with non-printed hydrogel–BBG
- 3D-printed hydrogel–BBG: Wounds covered with 3D-printed hydrogel–BBG dressings

**Figure 2.**
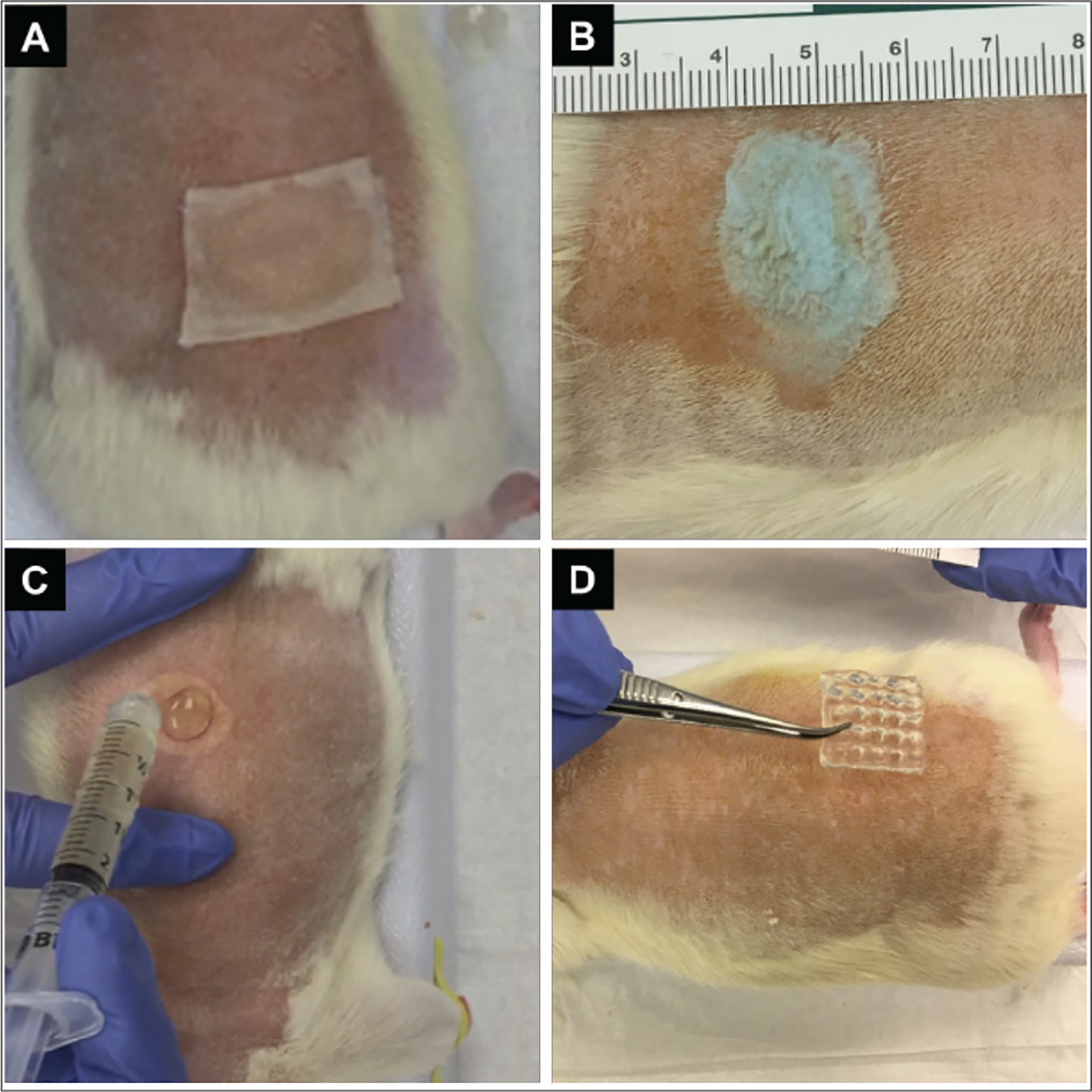
Animal test to evaluate second-degree burn wound healing using a rat model in six groups. Burn wounds covered with (A) petrolatum gauze, (B) BBG powder, (C) non-printed hydrogel and hydrogel–BBG, and (D) 3D-printed hydrogel and hydrogel–BBG dressings.

The animals were anesthetized using isoflurane. After shaving the dorsal area, the skin was cleaned with iodine and then sterilized with alcohol swabs three times. The second-degree burn was made by placing a 100°C aluminum bar with a diameter of 20 mm on the dorsal area for 10 s. After implementation, the wounds were disinfected by Dermoplast antiseptic spray (Advantice Health LLC, New Jersey, USA). After applying the dressings, the wounds were covered with Petrolatum Gauze and Elastikon bandages (3M, Minnesota, USA). Figure 2 shows the application of dressing on the wounds in the three groups. All animals were monitored for post-operative care on a daily basis. The wounds were assessed and photographed under isoflurane every 7 days. Prior to rebandaging, any necrotic tissue present on the wound surface was removed using sharp debridement if needed. Sharp debridement was performed by a trained medical professional using sterile surgical instrument, following established protocols. Necrotic tissue was defined as non-viable tissue that appeared black, brown, or gray in color and had a dry, leathery texture. The extent of necrotic tissue removal was documented for each sample. The animals were euthanized after 4 weeks using a lethal dose of CO_2_. Wound tissue explants were collected and fixed in formalin solution overnight for further histology investigation.

#### 2.9.2. Wound closure

Wounds were rebandaged and photographed every 7 days to track the wound size, color, edge, re-epithelialization, necrotic tissue formation, and secondary trauma caused by dressing removal. A sterile disposable ruler was placed in close proximity to the wound, serving as a scale for measurement purposes. The wound size was quantified by tracing the wound border in each photograph using ImageJ software. The wound closure was calculated as follows:

(V) $$\text{Wound}\;\text{closure}(\%)=\frac{{\text{A}}_{0}-{\text{A}}_{\text{w}}}{{\text{A}}_{0}}\times 100$$

where *A*_0_ is the wound area after wound creation, and *A*_*w*_ is the wound area at time t (*i.e*., 1, 2, 3, and 4 weeks). Traumatic removal was evaluated by assessing the presence of traumatic laceration, bleeding, and redness in wound margins and surrounding tissues after the dressing removal.

#### 2.9.3. Histology analysis

Full-thickness wound tissue explants (25 × 25 mm^2^) were resected and fixed overnight in 10% neutral buffered formalin, then cut into tissue blocks (25 × 1 mm^2^) that include wound bed, margins, and surrounding skin. Tissue blocks were processed and paraffinized using a fully automated tissue processor (TissueTek 2000, Sakura Finetek, California, USA). Tissue blocks were sectioned at 5 μm thickness and stained with hematoxylin and eosin (H&E). The slides were imaged using a transmitted light bright field microscope (Olympus BX53 microscope fitted with an Olympus DP70 digital camera) with a 10× objective lens. The entire tissue sections were scanned, digitally photographed, and “stitched” together to form a single composite image using Adobe Photoshop (Adobe Inc., California, USA). Quantitative histomorphometry was performed to measure the epidermal layer, dermal layer, and granulation tissue thickness. H&E images were blindly graded by two trained graders with sections scored on a scale of 0–4 regarding re-epithelialization, dermal regeneration, and granulation tissue formation^[65]^, as described in Table 1.

### 2.10. Statistical analysis

In this research, all experiments were conducted with a minimum of five replications for each sample per test. All data were reported as the mean ± standard deviation (SD). One-way analysis of variance (ANOVA) was employed to determine statistical difference among different groups. This statistical test enables the evaluation of overall group differences and determines if there are significant differences between the means of these groups. Following the ANOVA, the Student’s *t*-test is performed for pairwise comparisons between specific groups. This test allows for the determination of statistically significant differences between the means of two groups. Significance was set at the P-value <0.05. Statistical analyses were performed using GraphPad Prism 9.

## 3. Results and discussion

### 3.1. Mechanical properties

**Figure 3.**
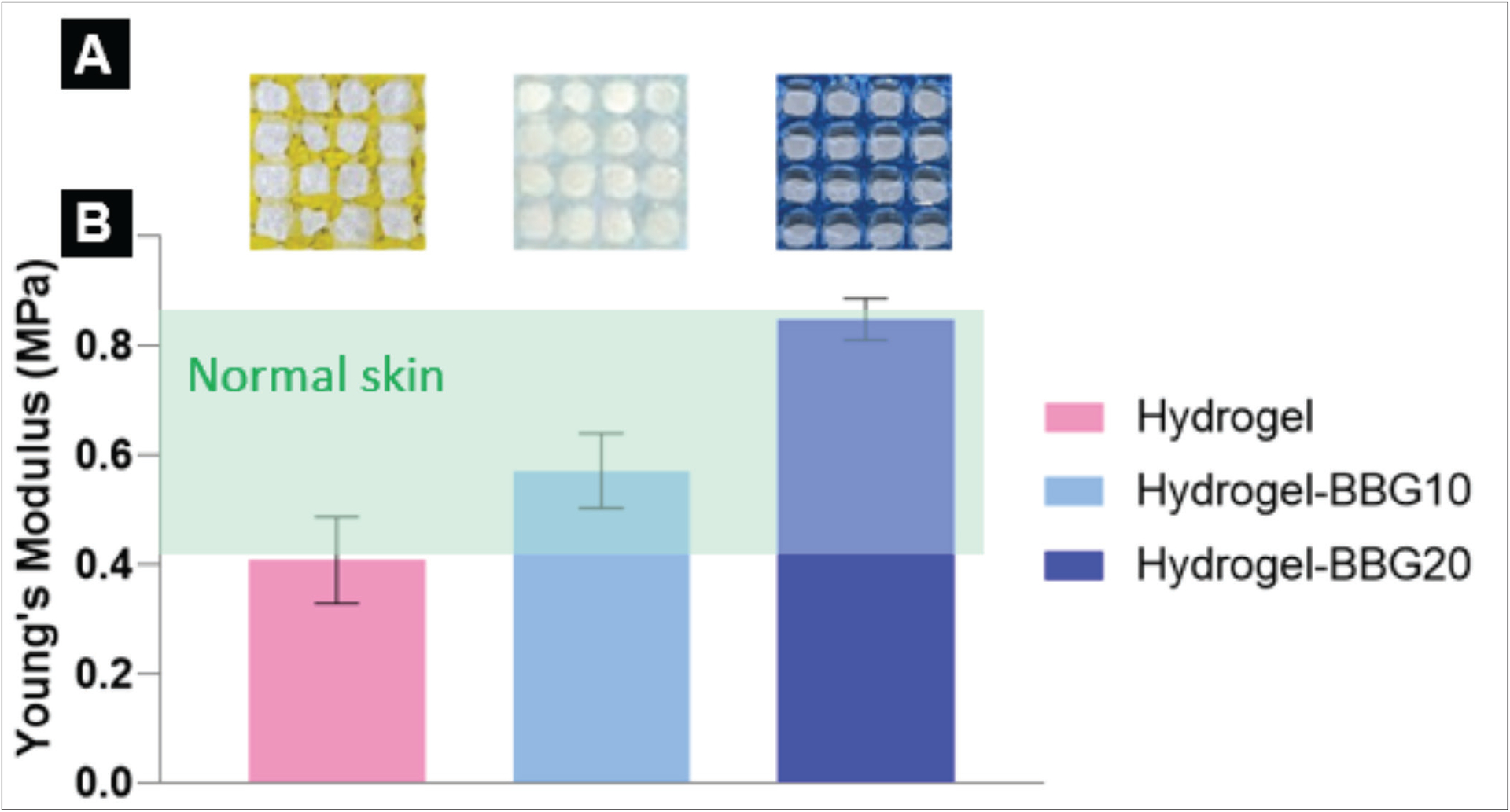
BBG improved the printing outcome, consistency, and Young’s modulus of 3D-printed dressings. (A) Photographs of the 3D-printed dressings. Hydrogel–BBG20 dressings showed the finest mesh structure and best shape fidelity. (B) The Young’s modulus of the 3D-printed dressings, compared to the normal unwounded skin. Both samples with BBG exhibited Young’s modulus in the range of normal skin (*n* = 5). The Young’s modulus of the normal skin is adopted from^[67,68].^

The 3D-printed hydrogel–BBG dressings were fabricated using extrusion-based 3D printing technology with different percentages of BBG content. Figure 3 shows the appearance and Young’s modulus of the 3D-printed dressings after crosslinking with calcium ions. Traditionally, mechanical properties, such as bone scaffolds, are considered key characteristics for load-bearing tissue engineering scaffolds. However, the mechanical behavior of skin substitutes and wound dressings has become an increasingly important focus of preclinical studies. Mechanical properties can significantly impact the performance and clinical outcomes of these products, including level of pain and trauma experienced during application, coverage, and removal. In order to promote moist wound healing, the dressings are required to exhibit adequate mechanical properties to integrate with the wound bed and surrounding tissue, while simultaneously providing effective coverage against external pathogens and traumas. Also, dressings should exhibit elasticity in the range of surrounding skin to support the body movement and normal activities without pain and falling apart^[66]^. The Young’s modulus (*E*) of normal skin fluctuates between 0.42 MPa and 0.85 MPa^[67]^ and has the highest value of approximately 1 MPa^[68]^. The tensile testing results from this work strongly support the positive effect of BBG on the elasticity of the 3D-printed dressings: (i) stronger chemical bonds between BBG particles and alginate chains and (ii) reinforcement of hydrogel network with BBG particles. It is generally accepted that the stronger chemical bonds in the hydrogel network result in higher mechanical strength and lower permeability. By increasing the amount of BBG from 0 to 10 and 20 w/v%, Young’s modulus increased by 39% and 105%, respectively.

### 3.2. Chemical structure

**Figure 4.**
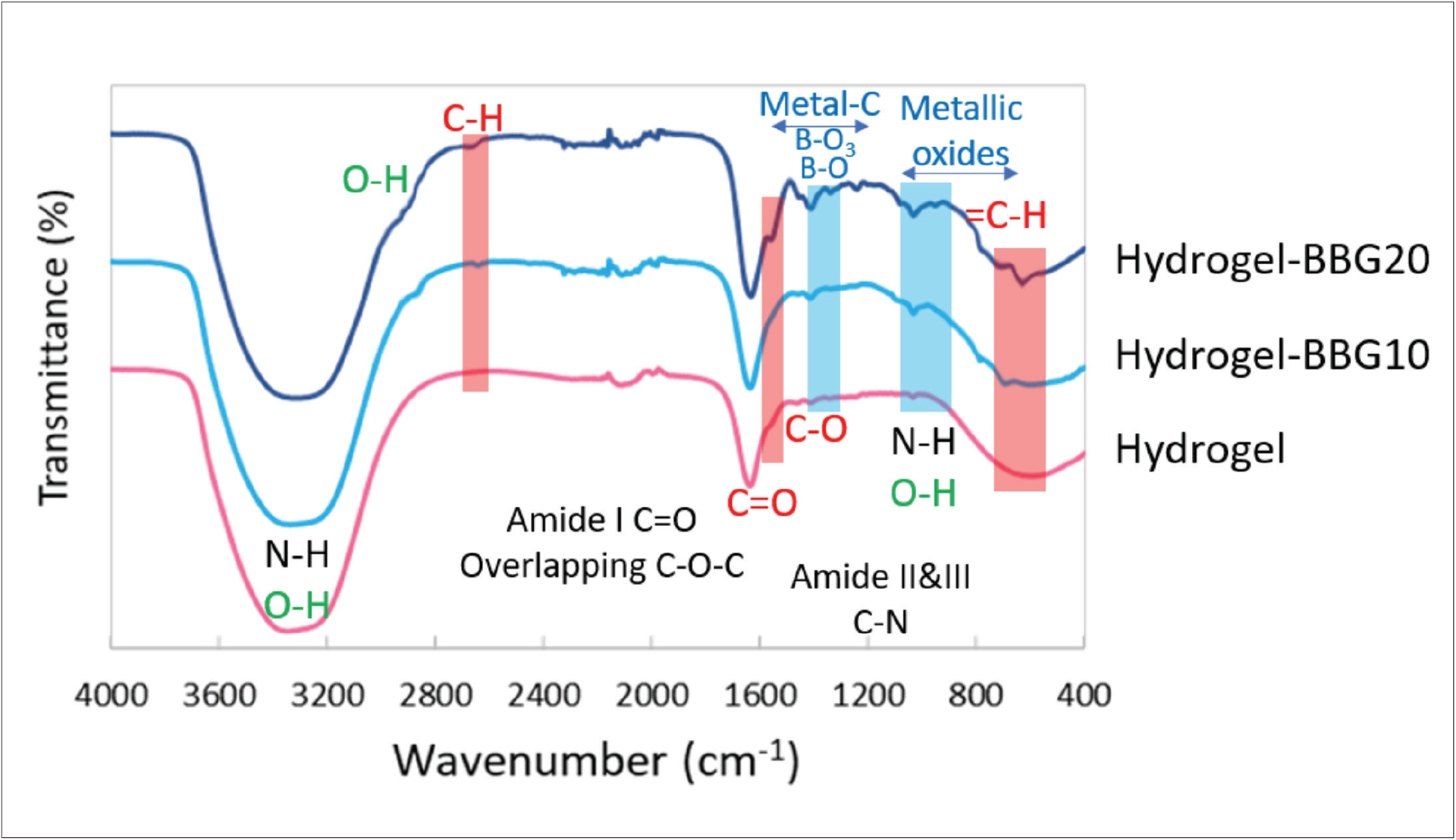
Fourier-transform infrared spectroscopy spectra of the hydrogel–BBG blends. The characteristic IR bands associated with BBG, gelatin, alginate, and water are shown by blue, black, red, and green text/boxes, respectively. BBG increased the intramolecular hydrogen bonds and formation of crosslinks between alginate chains.

Typically, the hydrogel-based bioinks provide favorable permeability to oxygen and nutrients. In order to evaluate the interactions between the gelatin, alginate, and BBG particulates, the chemical structure of the samples was studied by FTIR spectroscopy, and the resulting spectra are presented in Figure 4. Alginate and gelatin have overlapping carboxylate groups and hydroxyl groups with 3200–3500 cm^−1^ characteristic peaks, which is also overlapping with O-H stretching bonds in water. The amide I, II, and III bonds in gelatin and C=O bond as the characteristic bond of alginate were observed in all samples at a narrow peak at 1659–1243 cm^−1^ bands^[16]^. By increasing the BBG content, the intensity of hydrogen bonds decreases due to the formation of crosslinks that show the hydroxyl groups in alginate are involved in crosslinks with divalent metals instead of hydrogen bonds. It can be a result of the interactions between bivalent metals (*e.g*., Ca^2+^, Mg^2+^, Cu^2+^, Zn^2+^) released from BBG and alginate and the formation of crosslinks^[69]^. Bands associated with alginate showed significant changes by adding BBG. The bands associated with guluronic acid groups appeared between 500–450 cm^−1^ and 821 cm^−1^ were increased by BBG content, which shows the formation of =C-H groups. By increasing the BBG content, peaks associated with B-O and B-O_3_ appeared at 1402 cm^−1^ band^[70,71]^, which increased in hydrogel–BBG20 compared to hydrogel–BBG10. The peaks associated with metallic oxides within BBG, such as Ca-O, Mn-O, Cu-O, and Zn-O, appeared at 1000–600 cm^−1^ bands^[72–76]^. The C-O-C stretching bonds at 1080–1030 cm^−1^ and C=O bonds at 1621 cm^−1^ in guluronic acid increased by BBG, which confirms the formation of crosslinks in alginate in the presence of ions released from BBG^[69]^. The C-H stretching bands at 2600 cm^−1^ slightly appeared in hydrogel–BBG10 and increased in hydrogel–BBG20, confirming the interactions between ions released from BBG and gelatin chains, and the formation of crosslinks within alginate chains. The slight increase in O-H bonds at 2901 and 1021 cm^−1^ shows the increase in hydrogen bonds and interactions between water molecules and ions released from BBG. The appearance of peaks at 1630 cm^−1^ can be attributed to the reactions between proton donator groups in gelatin amide groups and the cations released from BBG. The formation of intermolecular hydrogen bonds between water molecules, ions, and different overlapping functional groups in gelatin and alginate makes a favorable entanglement for enhanced mechanical behavior at certain ratios of hydrogel:BBG. In the same line with mechanical testing results, forming covalent crosslinks between bivalent ions and guluronic acid blocks in alginate results in lower permeability and higher mechanical stiffness in samples with higher BBG content. Lower permeability reduces the transport of water molecules and maintains the structural integrity and stability of the hydrogel over time. The higher degree of crosslinking between alginate and BBG content can slow down the release of water which is desired for treatment of burn wounds.

### 3.3. Degradation rate and stability

**Figure 5.**
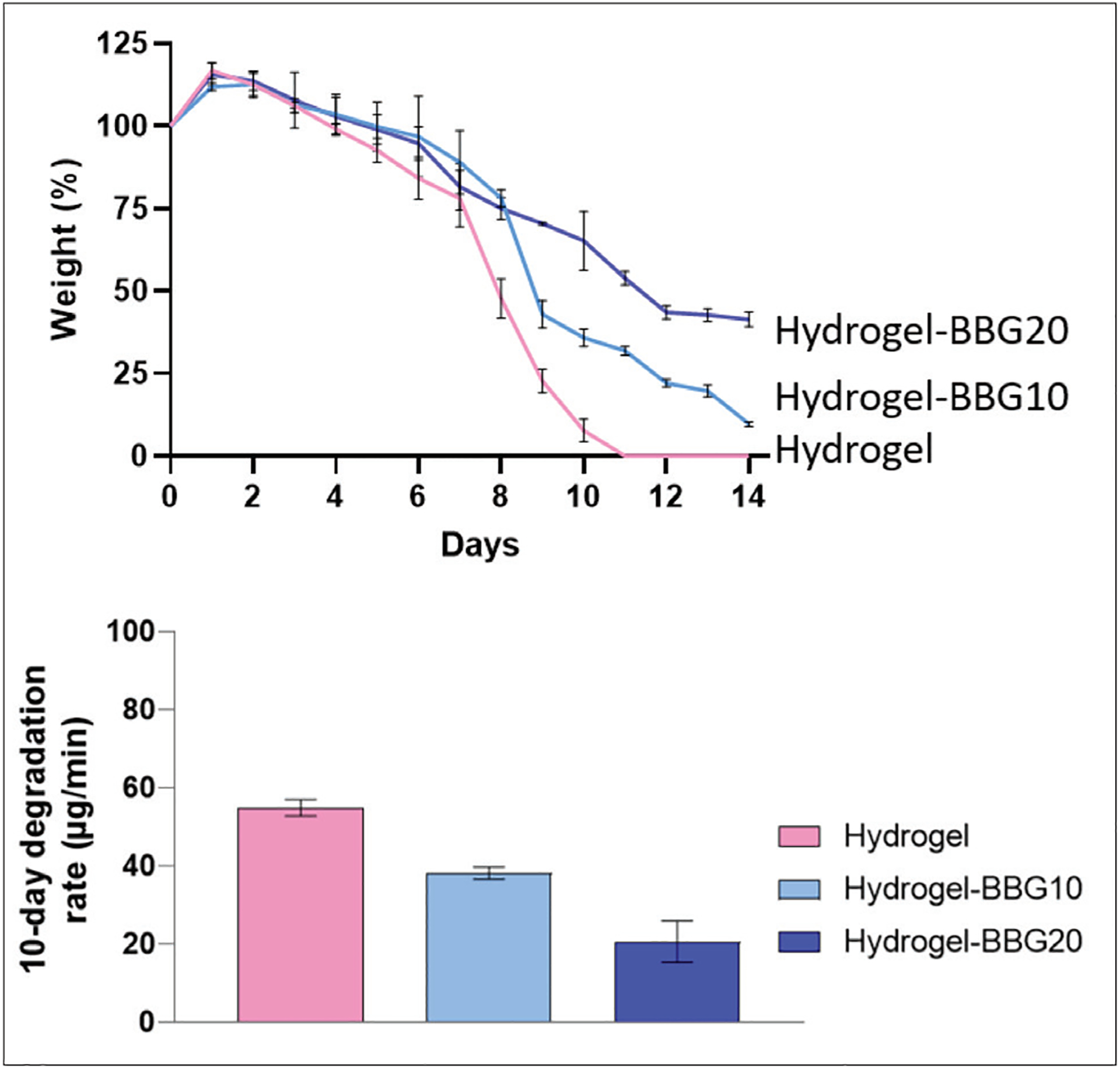
The swelling capacity, degradation profile, and 10-day degradation rate of the 3D-printed dressings in PBS (*n* = 5). BBG did not significantly affect swelling capacity (*P* >0.05), but it decreased the degradation rate. Samples with higher gelatin content showed a faster degradation rate and higher swelling capacity. BBG also increased the stability of the hydrogel: the 3D-printed hydrogel dressings without BBG degraded at day 10, whereas the BBG–hydrogel dressing lasted for 14 days. BBG particulates decreased the permeability of the hydrogel.

The swelling/weight change and degradation rate of the 3D-printed dressing are shown in Figure 5. The addition of BBG has negligible influences on the hydrogels and swelling capacity, while the 10-day degradation rate decreased by 29% and 62% after adding 10 and 20 w/v% BBG, respectively. BBG improved the stability of the hydrogel by increasing the degradation time from 10 to 14 days. It can result from (i) stronger electrostatic interactions, including hydrogen bonds and van der Waals bonds between BBG and hydrogel chains and (ii) covalent crosslinks between alginate chains in the presence of ions released from BBG. As illustrated in Figure 1, adding BBG decreased the free volume within the hydrogel network, increasing the density and decreasing the samples’ permeability. The crosslinking process of sodium–alginate results from Na–Ca replacement and formation of the egg-box structure. Since each Ca^2+^ ion can bond to two carboxylate groups, the ions can crosslink the polymer chains, which results in the formation of an insoluble, gel-like substance. This is associated with the presence of guluronic acid blocks, as shown in Figure 4.

### 3.4. Hydration activity and water release kinetics

**Figure 6.**
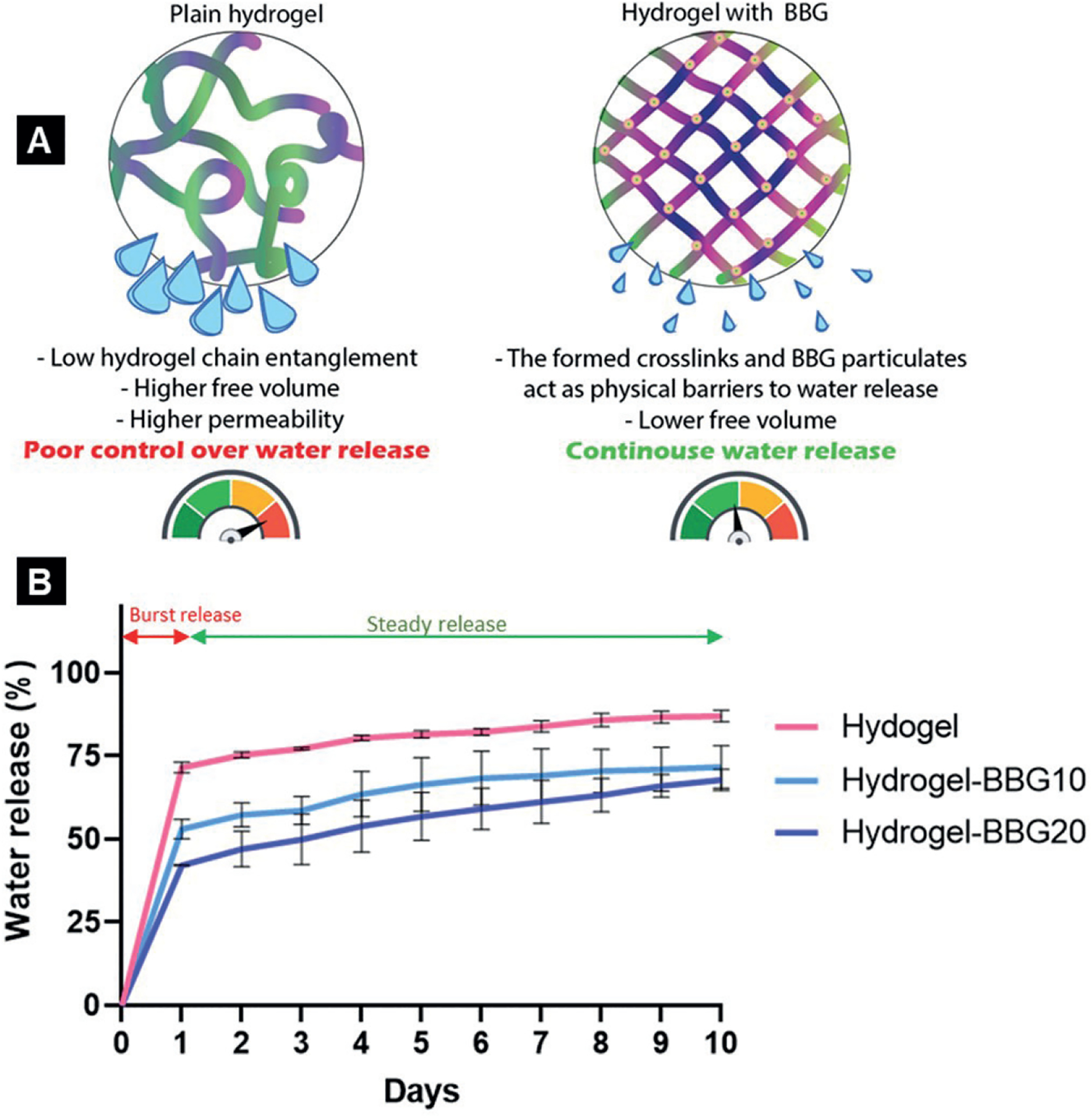
(A) Schematic of the effect of BBG on water release. (B) Ten-day hydration activity of the 3D-printed dressing on a super-absorbent surface to simulate dry burn wound surface (*n* = 5). BBG improved the water release by decreasing burst release and increasing the long-lasting water release. BBG decreased the permeability in the hydrogel, which justifies its slower degradation rate and sustained water release.

We studied the water content and water donation ability of the 3D-printed dressings to predict their functionality for clinical burn wound treatment. The initial water content slightly decreased by adding BBG content, as 3D-printed hydrogel, hydrogel–BBG10, and hydrogel–BBG20 dressings showed 94.36 ± 0.29, 94.01 ± 0.09, and 93.71 ± 0.24% water content, respectively. Figure 6 depicts the 10-day water release from the dressings on ethylcellulose substrate as a super-absorbent surface representing the dehydrated surface of burn wounds. It results from lower free volume in samples with higher BBG content and stronger chemical bonds. On the other hand, the crosslinking reaction between alginate chains and bivalent ions is a condensation reaction that releases water to build larger molecules^[77]^. Hence, increasing the degree of crosslinking decreases the amount of water entrapped within the hydrogel network. Adding BBG decreased the water release rate and prevented the burst release of water content in the first day. The overnight water release decreased by 25% and 42% in samples with 10 and 20 w% BBG compared to the plain hydrogel. The slower water release is a key factor for continuous water release and burn wound treatment outcomes, as the dressing can stay effective on the wound for a longer time, which decreases pain and infection risk associated with rebandaging, as well as the treatment costs. BBG particulates can act as physical barriers to water molecules’ movement; hence, the water molecules require higher energy and longer time to escape the hydrogel network.

To evaluate the efficiency of the hydrogel–BBG complex as a carrier for the controlled release of water, the release kinetics was analyzed using the *in vitro* cumulative water release data (Figure 6) at 32°C for 10 days. The graph was fitted with different kinetic models in accordance with square root values, as shown in Table 2. The Higuchi model was selected as the best-fitted release kinetic model for all samples, which implies that the kinetic of water release from 3D-printed hydrogel, hydrogel–BBG10, hydrogel–BBG20 dressings, and SA/Pec/TA-Ag nanocomposite follows the Higuchi square root model. This model allows for quantifying drug release from thin ointment films, hydrogel scaffolds, transdermal patches, and matrix devices over the skin^[78,79]^, which perfectly matches our 3D-printed dressings as porous hydrogel scaffold indicated for dermal contact and wound healing.

As shown in Figure 6, the release profile for all samples started with an initial burst release and was followed by sustained release, *i.e*., steady release. As shown in Table 2, BBG improved the 10-day water release by (i) decreasing the initial burst release and (ii) increasing the sustained water release rate. It means the 3D-printed dressings with 20 wt% BBG can gradually release more water per day. In contrast, the plain hydrogel dressing releases the majority of the entrapped water at the first day, instead of gradual release.

### 3.5. Cell viability

**Figure 7.**
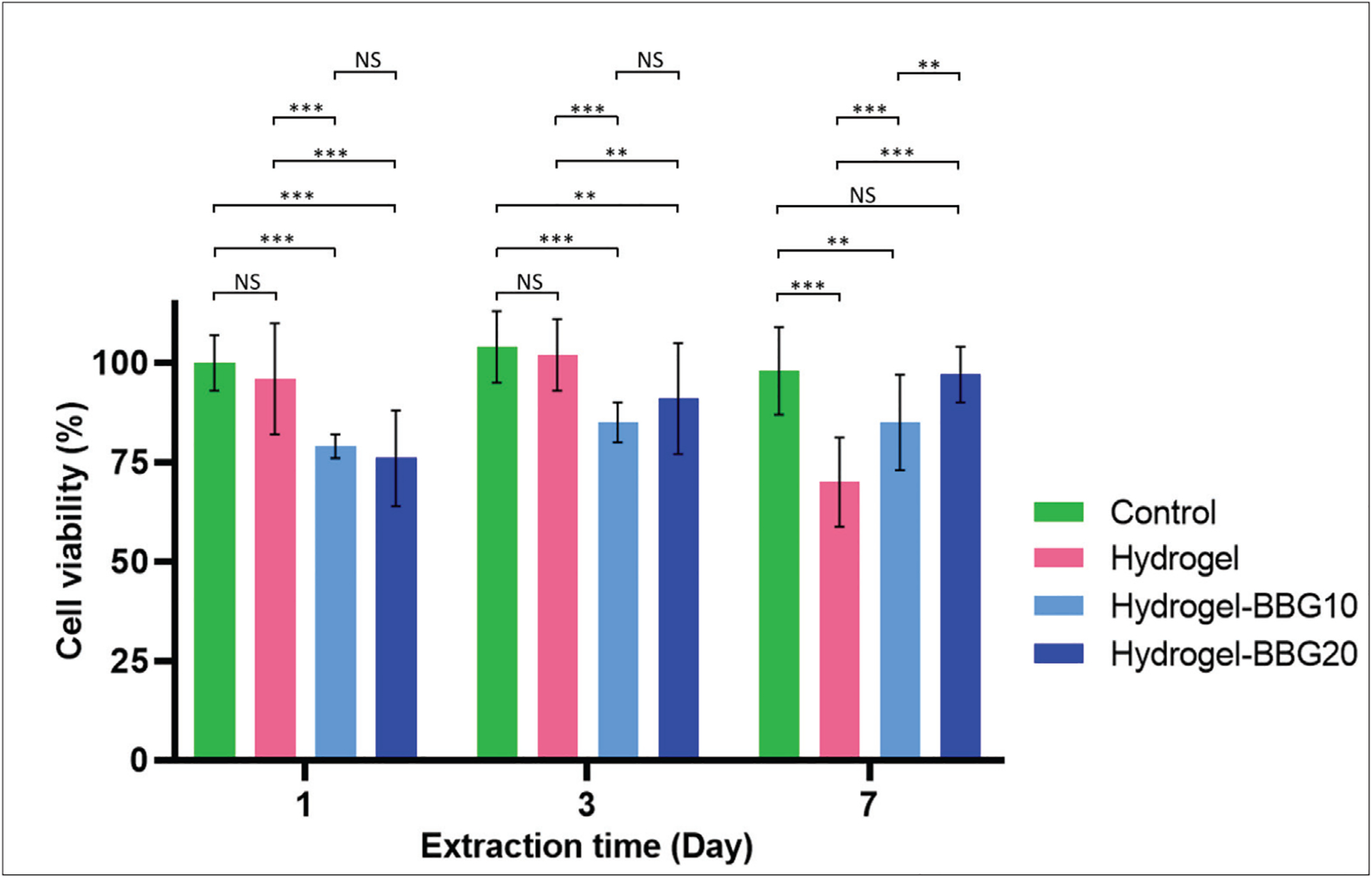
*In vitro* cell viability of the 3D-printed dressings using primary human dermal fibroblast after 1, 3, and 7 days of exposure. Hydrogel samples showed higher cell viability on days 1 and 3, and a decline on day 7 compared to samples with BBG showing lower cell viability. While BBG increased the cell viability after 7 days, which indicates the long-term effect of therapeutic ions released from BBG. In hydrogel samples, the short-term higher cell viability can be a result of RGD sequences present in gelatin^[80].^ *n* = 6; *P < 0.05, **P < 0.01, ***P < 0.001, and NS denotes non-significant difference.

The *in vitro* biocompatibility of 3D-printed dressings was evaluated by the MTT assay using primary human dermal fibroblasts (HDF) (Figure 7). 3D-printed hydrogel dressings showed significantly higher cell viability on days 1 and 3, and a decline on day 7 compared to the samples with BBG. The 7-day extracts from 3D-printed hydrogel–BBG20 showed the highest cell viability compared to the 3D-printed plain hydrogel and hydrogel–BBG10 (*P* < 0.05, *n* = 6), with no significant difference with the control group (*P* > 0.05). The decline in 7-day cell viability of the hydrogel dressings (without BBG) can be associated with the time-dependent denaturation of amino acid sequences in gelatin and acidic degradation of alginate. These results show that the biocompatibility of the samples is time-dependent and promoted by increasing the cumulative therapeutic ions released from BBG while negatively affected by the timely decomposition of the gelatin–alginate compound. The ion released from BBG shifts the pH to alkaline ranges, while the hydrogel degradation shifts the pH to acidic ranges. The improved cell viability in hydrogel–BBG20 samples indicates that the BBG content dominantly affects cell viability. In the same line, the reduced cell viability in hydrogel–BBG10 samples compared to hydrogel–BBG20 samples shows that in this sample, the cell viability is mainly affected by the adverse interactions between alkaline ions and acidic residues resulting from the decomposition of alginate. Hence, the hydrogel–BBG20 samples provide a favorable balance between the alkaline pH caused by ion released from BBG and the acidic degradation of alginate. On the other hand, the neutralized pH can preserve the arginine-glycine-aspartic acid (RGD) sequences for a longer time and enhance cell proliferation and growth^[80]^.

### 3.6. Animal test

**Figure 8.**
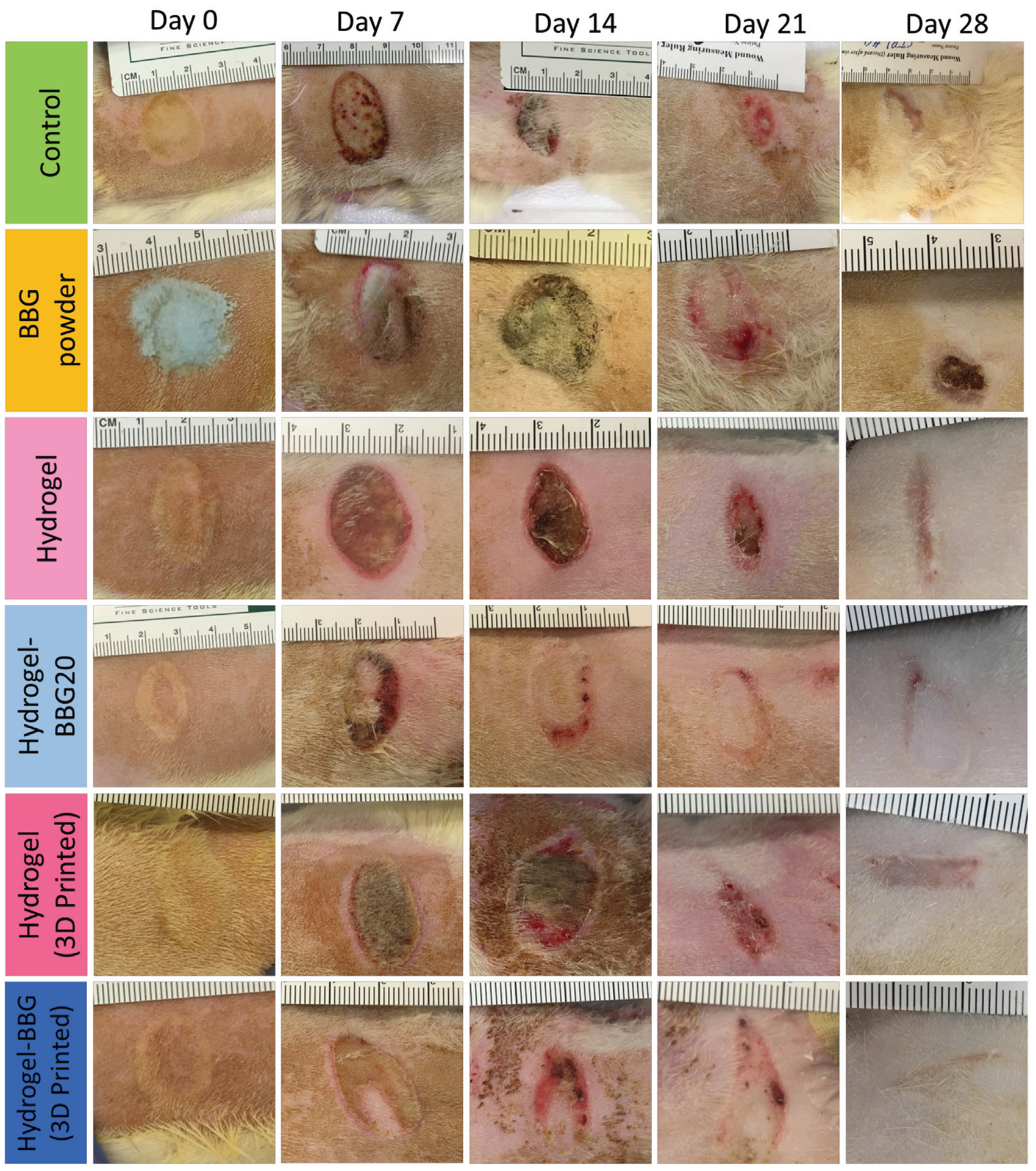
Gross examination of wound healing over 28 days (*n* = 6). Wound images from the control and treatment groups. 3D-printed and non-printed hydrogels with BBG showed earlier re-epithelization, less necrotic tissue, and smoother wound margins. In contrast, the 3D-printed dressings with porous contact surface support the non-adhesive removal of wound dressings.

**Figure 9.**
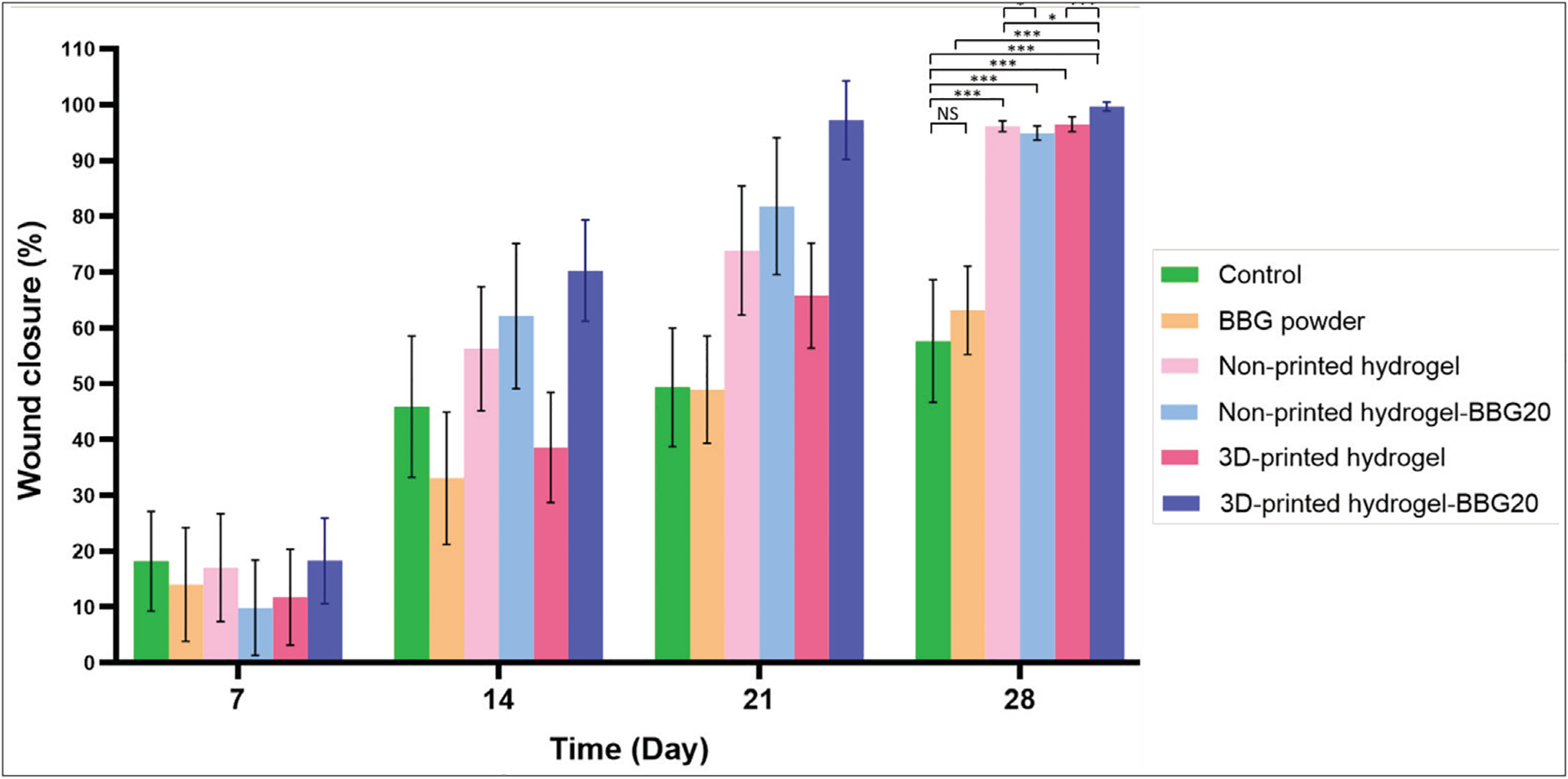
*In vivo* burn wound closure of the dressings in the control and test groups. 3D-printed dressings with BBG showed the fastest wound closure (*i.e*., smaller wound size) after 28 days, followed by 3D-printed hydrogel and non-printed hydrogel–BBG dressings. *n* = 6; *P < 0.05, **P < 0.01, ***P < 0.001, and NS denotes non-significant difference.

To assess the effect of BBG on the wound-healing activity of the 3D-printed dressings, we conducted an *in vivo* study using a rat model with a second-degree burn wound. We compared the wound closure time, re-epithelialization, and granulation tissue formation in the 3D-printed hydrogel and hydrogel–BBG20 dressings with the non-printed hydrogels of the same formulation. The as-received BBG powder and commercial petrolatum gauze served as control groups. Wound images on days 0, 7, 14, 21, and 28 were analyzed to estimate the wound contraction/closure ranging from the initial deep partial-thickness burn of 20 mm diameter on day 0 to the total wound closure on day 28. Figures 8 and 9 present the changes in wound appearance and area, *i.e*., wound closure, as a key factor in wound assessment. All samples showed faster wound closure compared to the control and BBG powder groups. Despite the excellent outcomes for chronic wounds, BBG has no therapeutic effect on burn wounds, which is due to the absence of aqueous media for therapeutic ion release and transfer to the wound. These results also re-affirm the positive effect of water release and hydrogel coverage on burn wound healing. Among the hydrogel samples, 3D-printed hydrogel–BBG20 showed the fastest wound closure and earliest re-epithelialization (*P* < 0.05, *n* = 6). Both 3D-printed and non-printed dressings with BBG showed smooth wound margins compared to the plain hydrogel samples with uneven wound margins. In contrast, the BBG powder group showed the formation of thick scab layer, *i.e*., dry and rough wound crust. The 3D-printed samples with and without BBG showed significantly faster wound closure than the non-printed dressings of the same composition.

Faster wound healing reduces the burden of second-degree burn wound by allowing the patients to resume their daily activities with shorter recovery time. The presence of an open wound is associated with pain, discomfort, and secondary trauma. Additionally, impaired or prolonged wound closure in second-degree burn wounds increases the risk of bacterial colonization and subsequent infection. Thus, faster wound healing contributes to patients’ quality of life by reducing pain, discomfort, and complications such as infection^[81,82]^.

Table 3 compares different parameters of wound healing recorded during weekly wound assessment. None of the samples developed an infection or adverse inflammatory response. The thickest and largest necrotic tissue was seen in the BBG group, followed by the control group. Both samples required sharp debridement for the removal of necrotic tissue. The sharp debridement as an invasive procedure slows the healing time and results in significant pain with further analgesia administration. In contrast, the 3D-printed hydrogel and hydrogel–BBG20 dressings and non-printed hydrogels of the same formulations developed smaller necrotic tissue and enabled autolytic debridement, *i.e*., the non-invasive spontaneous removal of necrotic tissue. The dressing removal in the BBG powder and control groups required intensive force and sharp instruments that caused severe damage to the fragile wound bed and surrounding tissues due to the adherence of the wound to the dressing surface. In the non-printed hydrogel and hydrogel–BBG20 groups, the wounds were thoroughly rinsed prior to rebandaging to detach the dressing residues from the wound surface non-invasively. The porous contact in 3D-printed hydrogel and hydrogel–BBG20 dressings allowed for the easy and atraumatic removal of these dressings from the wound surfaces with no pain or damage to the granulation tissue or re-epithelialization layer. Unlike the non-printed hydrogel, BBG powder, and control groups, the 3D-printed hydrogel and hydrogel–BBG20 dressings and non-printed hydrogel–BBG dressings showed smooth wound margins and optimal wound closure, which can be attributed to (i) the bioactive formulation and continuous hydration in the 3D-printed and non-printed hydrogel–BBG20 groups, which promote the moist wound healing for longer time periods and (ii) the non-adhesive contact and porous texture of the 3D-printed dressings, which increase the available surface for wound-dressing interactions. The poor wound healing activity in the non-printed hydrogel dressings (without BBG) may result from the fast and excessive water release, which causes early wound maceration (*i.e*., excessive water absorption in the wound and surrounding tissues) with long-term wound dehydration^[82,83]^. As shown in Figures 6 and 8, the poor control over water release in the non-printed samples results in inadequate wound hydration during the 7-day wound coverage. The dry surface of the BBG powder and petrolatum gauze in the control group can only protect the wound from infection and water loss due to evaporation, which results in wound dehydration and prolonged healing.

**Figure 10.**
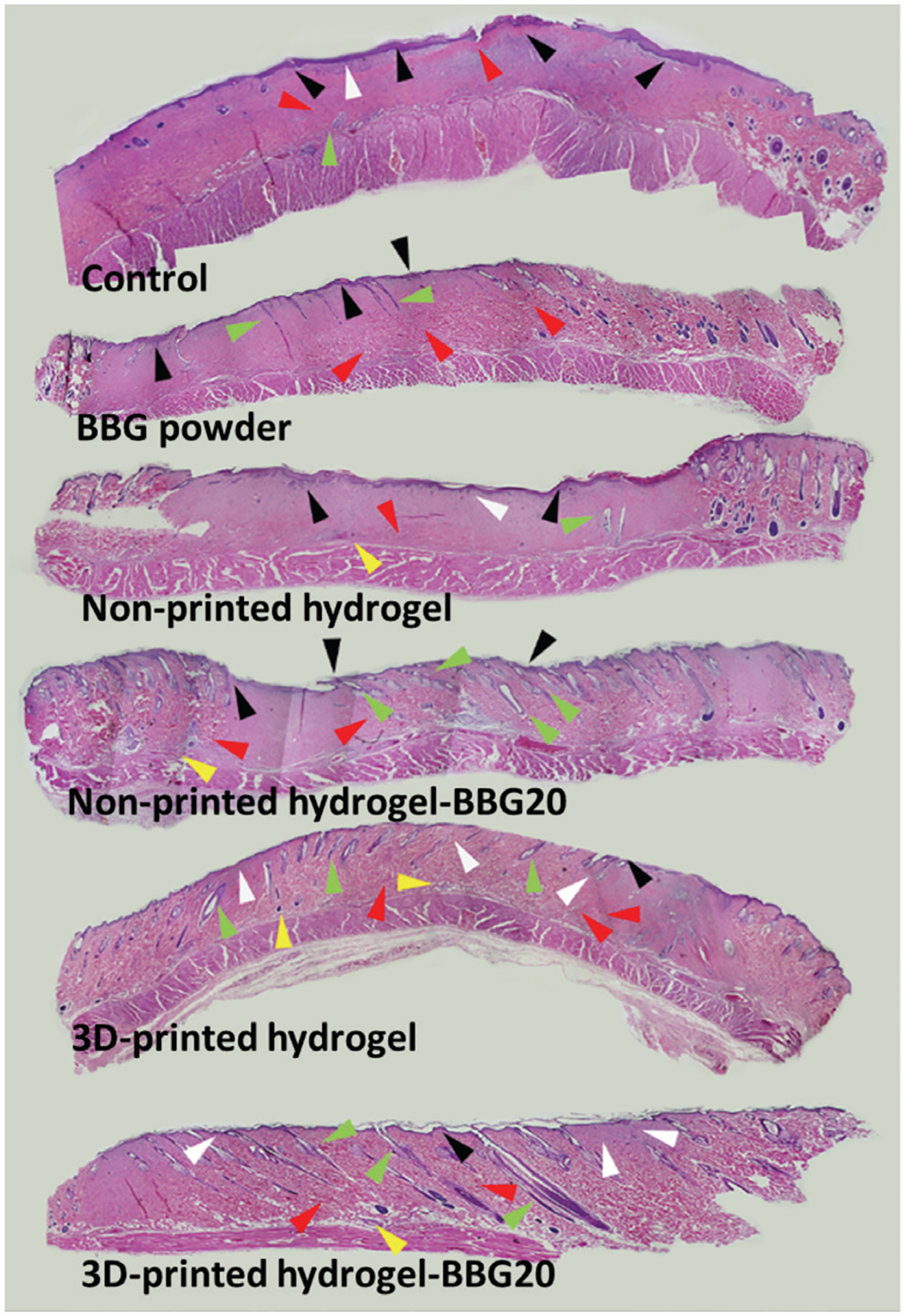
Representative the wound tissues stained with H&E at 10× magnification. 3D-printed dressing composed of hydrogel–BBG20 accelerated wound healing and guided the regeneration of hair follicles in second-degree thermal burns in a rat model. BBG guided wound healing to normal wound closure rather than wound contracture by promoting formation of a uniform epidermal layer, regeneration of hair follicles, and mature granulation tissue formation. Compared to the non-printed samples with the same formulation, the 3D-printed dressings with and without BBG improved the uniform regeneration of the dermal layer. Guides labeled in the figure: hyperkeratosis (black arrowhead), epidermal regeneration (outmost layer in dark purple), dermal layer (white arrowhead), granulation tissue (red arrowhead), hair follicle (green arrowhead), and sweat glands (yellow arrowhead).

Figure 10 shows representative H&E-stained slides obtained from different groups to investigate epidermal regeneration (ER), dermal regeneration (DR), and granulation tissue formation (GT), which are key factors in wound healing analysis. The control and non-printed hydrogel groups showed the thickest ER layer, *i.e*., hyperkeratosis, which is associated with poor skin regeneration. The non-printed hydrogel–BBG20, 3D-printed hydrogel, and 3D-printed hydrogel–BBG20 dressings developed thicker epidermal layer. In contrast, both 3D-printed dressings showed even ER, which indicates the positive effect of the porous texture of the 3D-printed contact on tissue regeneration. Despite the poor wound healing, the BBG powder group showed slight regeneration of hair follicles. The non-printed hydrogel–BBG20 and 3D-printed hydrogel–BBG20 showed the highest regeneration of hair follicles, which shows the significant effect of BBG on post-burn regeneration of hair follicles. The distinctive regeneration of hair follicles in these samples can be due to the continuous hydration and non-adhesive surface with aligned pores. The 3D-printed samples, regardless of the formulation, increased the regeneration of skin appendages by providing a favorable interface for cell–material interaction within the pores. The non-printed hydrogel–BBG samples, 3D-printed hydrogel, and hydrogel–BBG dressings showed higher numbers and faster regeneration of hair follicles compared to the other samples. The non-printed and 3D-printed hydrogel–BBG20 dressings developed thicker and more hair follicles toward the epidermal layer, while in the BBG powder, non-printed hydrogel, and 3D-printed hydrogel groups, the hair follicles were shorter, and still in the dermal layer, indicating slower growth and development of hair follicles. Therefore, the synergistic effect of BBG and 3D-printed porous surface enhanced the regeneration of follicles in terms of population and growth rate. The presence of GT after 21 days is a major indication of immature wound healing and prolonged tissue regeneration. Further, more sweat glands and skin appendages (yellow arrowheads) appeared in all groups, with slightly higher regeneration in the 3D-printed hydrogel–BBG20 group. The control group showed the highest GT on day 28, confirming poor wound closure in this sample (Figure 8). More specifically, GT refers to the chronically vascularized tissue that represents persistent inflammation, mainly composed of pink and granular tissue with macrophages and proliferating fibroblasts^[84]^. The persistence of GT at week 4 represents the immature wound healing and failed treatment. All samples showed slight GT formation compared to the control groups, showing that both 3D-printed porous texture and BBG content positively affected tissue regeneration. Recent research showed that a reduction in the formation of GT is associated with improved scar outcomes^[85]^. The 3D-printed samples with and without BBG showed uniform dermal regeneration that can be associated with the 3D-printed porous pattern in these samples. Our findings confirmed that the favorable degradation rate, controlled water release, skin-like mechanical properties, and porous non-adhesive contact surface in 3D-printed hydrogel–BBG20 dressings could promote the outcomes of burn wound healing.

Our results provide substantial evidence on the effects of BBG on mechanical properties, degradation rate, and hydration activity in 3D-printed gelatin–alginate dressings. The therapeutic ions released from BBG decreased the early cell viability of the samples. At the same time, the favorable interactions between the acidic degradation of alginate and the presence of RGD sequences from gelatin improved the 7-day cell viability of the samples. Electrostatic interactions between BBG particulates and hydrogel chains increase the stiffness and decrease the permeability of the dressings. The lower permeability in 3D-printed hydrogel–BBG20 slows down the degradation rate, increases the stability of the dressings, and regulates the water release rate from the hydrogel. Our findings showed that adding BBG to the gelatin–alginate compound enables controlled water release for up to 10 days, which is a key feature for burn wound healing. The kinetics of water release from 3D-printed dressings was fitted with the Higuchi model that refers to transdermal patches and hydrogel films. Accordingly, BBG content positively affected the *in vivo* wound healing outcomes in terms of dermal/epidermal regeneration and restoration of hair follicles in second-degree burn wound treatment. Overall, the addition of 20 wt% BBG promotes the functionality of 3D-printed hydrogel dressing by synergistic effect of continuous water release from 3D-printed dressings, favorable interactions between RGD sequences in gelatin, acidic degradation of alginate, and cumulative release of therapeutic ions from BBG.

## 4. Conclusion

In this study, we developed 3D-printed bioactive wound dressings using gelatin, alginate, and borate glass (BBG). The incorporation of BBG improved the tensile stiffness and cell viability of the 3D-printed dressings and regulated water release for maintaining optimal wound moisture. The safety and efficacy of the 3D-printed hydrogel–BBG dressings on second-degree burn wounds was assessed in a rat model. The 3D-printed hydrogel dressings incorporated with 20 wt% BBG showed faster wound closure and lower wound contracture compared to the non-printed hydrogel of the same composition, FDA-approved bioactive glass, and the standard of care. BBG content positively contributes to superb healing outcomes in the context of dermal/epidermal regeneration and hair follicle restoration. The clinical significance of incorporating BBG into 3D-printed hydrogel dressings lies in bioactive formulation, non-adhesive contact, and ability to maintain optimal wound moisture for up to 7 days. These features can potentially enhance the patient outcome by reducing the need for frequent dressing changes, minimizing the pain and the risk of infection, and promote faster wound closure.

The outcome of this study provides promising insights into using bioactive formulations for 3D printing as a versatile technology in tissue engineering and regenerative medicine. Our study has the potential to further research on more complicated bioinks incorporated with nanoparticles, signaling factors, and bioactive reagents to enhance the efficacy of bioactive hydrogel dressings. Advancements in fabrication methods and 3D printing of BBG, hydrogel, and other bioactive materials will contribute to the development of patient-specific dressings and skin substitutes, enabling customizable wound healing approaches. Therefore, the scalability and cost-effectiveness, as well as addressing the specific regulatory requirements of 3D-printed biomedical products, should be further advanced to ensure safe and effective deployment of these technologies in clinical settings. Another promising avenue in wound healing research is the advancement of stimuli-responsive dressings, *i.e*., smart dressings, capable of responding to specific physiological cues. For example, the pH-sensitive ion release and tunable water release from BBG–hydrogel dressings can be further investigated and combined with biosensors and 4D printing technologies to develop smart wound dressings that can rapidly detect infection and release therapeutic agents based on wound condition or environmental parameters (pH, temperature, oxygen level, moisture level, bacterial load, *etc*.). Another promising future direction in wound healing research is the integration of bioinformatics and artificial intelligence in wound monitoring, assessment, and documentation. The transparent appearance and programmable functionality of the hydrogel–BBG dressings can be combined with bioinformatics and artificial intelligence technologies to potentially enhance wound care by providing objective wound assessments, automating wound documentation, and facilitating data-driven decision-making. Finally, we envision that hydrogel–BBG bioinks can be extended by incorporating stem cells and other signaling factors to facilitate scarless wound healing and complex chronic ulcers and radiation burn injuries. Overall, our findings presented in this paper hold significant potential for improving patient outcomes and advancing the future research in the field of wound healing.

## Figures and Tables

**Table 1.** Qualitative histological grading criteria adopted from Altavilla *et al*.^[65]^

Score	Re-epithelialization	Dermal regeneration	Granulation tissue formation
0	No epidermal organization	No dermal organization	Very thin or no granular layer
1	Very little epidermal organization	Very little dermal organization	Thin granulation layer
2	Little epidermal organization	Little dermal organization	Moderate granulation layer
3	Moderate epidermal organization	Moderate dermal organization	Thick granular layer
4	Complete remodeling of the epidermis	Complete remodeling of dermis	Very thick granular layer

**Table 2.** Release kinetic models and parameters for water release from 3D-printed dressings

Release kinetics model	Hydrogel	Hydrogel-BBG10	Hydrogel-BBG20
Zero-order	R^2^ = 0.9494	R^2^ = 0.9183	R^2^ = 0.9815
First-order	R^2^ = 0.9826	R^2^ = 0.9446	R^2^ = 0.9960
Korsmeyer-Peppas	R^2^ = 0.7389	R^2^ = 0.7112	R^2^ = 0.6782
Higuchi^a^	R^2^ = 0.9891	R^2^ = 0.9696	R^2^ = 0.9983
Hixon Crowell	R^2^ = 0.5432	R^2^ = 0.5831	R^2^ = 0.7138
**Drug release parameters**			
Burst release (%)	71.51 ± 1.59	53.07 ± 2.87	42.14 ± 0.2
Total release (%)	87.09 ± 1.73	71.77 ± 6.44	67.86 ± 3.2
Sustained release (%)	15.58 ± 3.27	18.69 ± 3.82	25.79 ± 3.07
Sustained release/burst release	0.21	0.38	0.55
Release rate (mg/day)	1.73	2.07	2.85

a The water release kinetics in all samples demonstrate the highest coefficient of determination (R2 value) when fitted to the Higuchi model compared to other models..

**Table 3.** Wound assessment for different study groups

Dressing	Moist wound healing	Granulation tissue (days 14 and 21)	Re-epithelialization (days 21 and 28)	Wound margins
Control	Not seen	3–2	2–2	Red—Thick crust
BBG powder	Not seen	4–3	1–3	Brown—Thick crust
Non-printed hydrogel	Partially seen	3–2	2–4	Pink—Sloping
Non-printed hydrogel-BBG20	Seen	2–0	4–4	Light pink—Smooth, flat
3D-printed hydrogel	Seen	3–1	3–4	Pink—Smooth, flat
3D-printed hydrogel-BBG20	Seen	2–0	4–4	Light pink—Smooth, flat

## Data Availability

To make the raw data used in this work available to readers, we can provide it upon request via email. Interested readers can contact us directly at f.fba@mst.edu to inquire about accessing the raw data..
